# The Mediating Role of Mattering in the Relationship Between Perceived Islamophobia and Well-Being in a Group of Muslim Women Residing in Italy

**DOI:** 10.3390/bs15101338

**Published:** 2025-09-29

**Authors:** Cristian Di Gesto, Elisa Guidi, Giulia Rosa Policardo, Amanda Nerini, Camilla Matera

**Affiliations:** 1 Department of Psychology, Sapienza University of Rome, Via dei Marsi, 78, 00185 Rome, Italy; 2 Department of Theoretical and Applied Sciences, eCampus University, Via Isimbardi, 10, 22060 Novedrate, Italy; elisa.guidi@uniecampus.it (E.G.); giuliarosa.policardo@uniecampus.it (G.R.P.); 3 Department of Education, Languages, Interculture, Literatures and Psychology, University of Florence, Via San Salvi, 12, 50135 Florence, Italy; amanda.nerini@unifi.it (A.N.); camilla.matera@unifi.it (C.M.)

**Keywords:** social well-being, social and community psychology, mattering, perceived discrimination, Muslim women

## Abstract

This study examined the associations between perceived Islamophobia and social well-being among Muslim women living in Italy, focusing on the potential mediating role of societal mattering and interpersonal mattering with respect to family and friends. Drawing from community and social psychology perspectives, we hypothesized that perceived Islamophobia would be negatively associated with social well-being, and that this relationship would be mediated by perceived mattering. In total, 120 Muslim women completed validated measures of perceived Islamophobia, mattering, and social well-being. Generalized Linear Modeling indicated that the direct association between perceived Islamophobia and social well-being was marginally significant. However, significant indirect associations emerged through societal mattering and mattering to friends. Higher perceived Islamophobia was associated with lower societal mattering and mattering to friends, which in turn were positively associated with social well-being, whereas mattering to family did not mediate the association. These findings underscore the role of different forms of mattering in shaping social well-being among women belonging to a minority religious group. Results have implications for the development of interventions aimed at promoting perception of being important to one’s society and significant others among Muslim women, particularly in national contexts where anti-Muslim sentiments may threaten their well-being.

## 1. Introduction

The 2024 ISMU ETS Report on Migration (Valtolina & Menonna, 2025) highlights that among the foreign population residing in Italy, Muslims now account for nearly 30% of the total, representing the largest religious group among immigrants in the country when the divisions within Christianity (Orthodox Christians, Catholics, Evangelicals, Copts, and other Christian groups) are considered separately. In addition to Muslims with foreign citizenship, Italy is also witnessing a growing presence of Muslims with Italian citizenship; as a result, the country ranks among those with the highest Muslim populations in the European Union (EU) (Ciocca, 2022; Pew Research Center, 2017). In the European context, a recent survey conducted by the EU Agency for Fundamental Rights (FRA, 2024) involving 9604 participants who self-identified as Muslim from 13 EU countries, including Italy, revealed a significant increase in racial discrimination based on skin color, ethnic or immigrant background, and religion or religious beliefs—from 25% in 2016 to 35% in 2022. Although Muslim respondents in Italy reported some of the lowest levels of racial discrimination (22% in the year preceding the survey), they also exhibited the lowest awareness of anti-discrimination legislation (32%). Furthermore, the Sixth Report of the European Commission against Racism and Intolerance (ECRI, 2024) indicates that there have been cases of anti-Muslim violence in Italy, with Muslim women wearing the headscarves being the primary victims.

Islamophobia—defined as a violation of human rights manifested through racism, discrimination, and violence—appears to be on the rise in Western Europe and North America (De Nolf & d’Haenens, 2024; Rehman & Hanley, 2022). Despite its detrimental impact on the well-being of Muslims globally, Islamophobia remains insufficiently explored within academic research, particularly in the field of psychology (Rehman & Hanley, 2022). Moreover, scholarly work on Islamophobia tends to prioritize the analysis of its racial dimension over its gendered aspects, further underscoring the urgency of addressing gendered Islamophobia (Alimahomed-Wilson, 2020). In the context of Muslim women and girls in Western diasporas, the concept of gendered Islamophobia refers to specific forms of ethno-religious and racialized discrimination targeting Muslim women, rooted in Orientalist stereotypes that portray them as backward and oppressed (Zine, 2006). These representations have served to justify colonial domination over Muslims by framing European intervention as an emancipatory act for Muslim women. This form of epistemic violence and oppression operates at multiple levels—social, political, and discursive—denying Muslim women access to material advantages (Zine, 2006).

A recent systematic review on the consequences of Islamophobia highlights gender as the primary mediating factor, emphasizing that Muslim women constitute the social group most frequently subjected to Islamophobia (De Nolf & d’Haenens, 2024). For example, in the healthcare context, approximately one-quarter of Muslim women in a U.S.-based study (Murrar et al., 2024) reported being treated worse than non-Muslim people and routinely experienced negative treatment in that setting. Also in the workplace, Muslim women, particularly those who wear the veil, experience greater discrimination than women from majority groups (Ahmed & Gorey, 2023; Khattab et al., 2019). Certain human capital factors, such as language proficiency, length of residence, and educational qualifications, significantly influence the occupational outcomes of Muslim women (Khattab et al., 2019). Finally, a study conducted in the United States (US) (Steele et al., 2021) found that, for Muslim women—but not for Muslim men—experiences of discrimination were associated with concealing their religious identity in public. This behavior may stem from the higher discrimination faced by Muslim women and may be adopted as a strategy to avoid further discriminatory incidents (Steele et al., 2021).

The present study aims to investigate the relationship between perceived Islamophobia and social well-being, focusing on the psychological processes that may mediate this association. In particular, the study examines the role of mattering as a protective factor, hypothesizing a central mediating role. Adopting an ecological perspective (Kelly, 2010), the analysis considers different dimensions of mattering, both at the interpersonal and societal levels. Few studies have examined the mechanisms through which perceived Islamophobia relates to social well-being, and this research addresses this gap by considering different dimensions of mattering in a group of Muslim women residing in Italy.

### 1.1. Perceived Discrimination, Islamophobia, and Well-Being Among Muslim Women

Over the past thirty years, community psychology has increasingly focused on well-being, developing theoretical reflections, empirical research, and interventions aimed at its promotion (Esposito et al., 2021). In 1998, Keyes defined social well-being as comprising five dimensions such as social coherence (i.e., the perception of the quality, functioning, and organization of society, as well as an interest in understanding it), social integration (i.e., the evaluation of the perceived quality of the individual’s connection with the community and broader society), social actualization (i.e., the evaluation of society’s potential as expressed through the actions of its institutions and members), social contribution (i.e., the evaluation that one’s contribution is useful and valued by society), and social acceptance (i.e., the view of society based on the perceived qualities of others) (C. L. M. Keyes, 1998). Social well-being is fundamental to the mental health of immigrants, which significantly depends on positive functioning within the receiving society, unlike for native-born individuals, for whom social exclusion has a less significant impact (Bobowik et al., 2015).

Given that compromised well-being may constitute a risk factor for mental health (e.g., Wood & Joseph, 2010), it is crucial to examine its antecedents. As highlighted by a recent literature review, perceived discrimination is a significant risk factor for mental health, exerting a stronger immediate negative impact on externalizing and stress-related outcomes compared to those associated with well-being and self-regulated mental health (Emmer et al., 2024). Moreover, as emerged in a recent study involving refugees in Italy, positive intergroup relations are associated with greater satisfaction with life and psychological well-being, but these beneficial effects are attenuated by the presence of negative contact (Nerini et al., 2025).

A study on Muslim Arab Americans found no significant direct relationship between discrimination and well-being; however, discrimination had a negative indirect effect on well-being through decreased American identification and a positive indirect effect on well-being through religious identification (Hakim et al., 2018). Focusing on Muslim women, a study by Jasperse et al. (2012) found no direct relationship between perceived religious discrimination and psychological well-being, while the psychological and behavioral dimensions of Muslim identity moderated this relationship. Specifically, a strong psychological affiliation with Islam intensified the negative effect of perceived discrimination on well-being, whereas engagement in Islamic practices mitigated this negative impact. Qualitative findings further deepen the understanding of how discrimination impacts Muslim women’s identity and well-being. For instance, Alsaidi et al. (2023) interviewed Muslim Arab American women and revealed that they often experience discrimination at the intersection of multiple identities (e.g., gender, ethnicity, race, and religion) which contributes to internalized stereotypes, psychological distress, and racial trauma.

Participants also reported a shift in societal stereotypes, with Muslim women increasingly being perceived not as passive victims, but as complicit in extremism. Such stigmatization negatively influenced their self-concept and sense of safety, particularly among those wearing the hijab. Nonetheless, many women responded with active self-acceptance, community advocacy, and increased cultural pride, illustrating coping strategies that buffer the psychological impact of discrimination. These findings underscore the importance of investigating how perceived discrimination relates to Muslim women’s social well-being and of exploring the psychological factors—such as perceived significance and meaningful engagement across multiple relational and societal spheres—that may contribute to this association.

### 1.2. Mattering as a Mediator of Well-Being on Perceived Discrimination

The concept of mattering has gained increasing attention as a key element for promoting health, empowerment, and social justice. According to Prilleltensky (2020), mattering—the perception that one is significant and makes a difference—represents a fundamental psychological need that is essential for human thriving. In his framework of meaning-making, mattering, and thriving, he emphasizes that the promotion of mattering is central to fostering individual and collective well-being in contexts marked by oppression, exclusion, or marginalization.

Mattering is generally conceptualized as comprising interpersonal and societal dimensions (Rosenberg & McCullough, 1981). Interpersonal mattering refers to the sense of being important to close others (e.g., friends, family), whereas societal mattering reflects the belief that one is valued and can contribute meaningfully to society at large (Rosenberg, 1985). Both dimensions represent distinct, yet interrelated, sources of psychological resources and forms of validation that can buffer against the negative effects of stigmatization and discrimination (Matera et al., 2021; Pernice et al., 2017).

There is growing empirical support for the positive association between mattering and well-being across different populations. For example, a recent study by Paradisi et al. (2023) found that mattering significantly predicted psychological well-being and life satisfaction in a sample of ethnically diverse youth in Italy. Similarly, studies conducted among racial and ethnic minorities have demonstrated that the perception of mattering contributes to better mental health outcomes and greater resilience in the face of marginalization (Flett, 2022; Pousson & O’Laughlin, 2024). Although specific research on Muslim women remains limited, existing literature suggests that mattering may represent a relevant psychological mechanism through which experiences of discrimination are linked to individual well-being, particularly in contexts marked by social exclusion.

Two studies have conceptualized mattering as a mediating variable in the relationship between perceived discrimination and well-being. Pousson & O’Laughlin (2024) showed that higher levels of perceived discrimination were associated with lower perceptions of mattering, which in turn predicted greater depressive symptoms among LGBTQ+ youth. Similarly, Wight et al. (2015) found that mattering partially mediated the association between internalized gay ageism (i.e., a specific manifestation of perceived discrimination combining elements of ageism and homophobia) and depressive symptoms among midlife and older gay men. According to the authors, experiences of perceived discrimination undermine individuals’ sense of being valued and significant within their social context, thereby weakening a key psychological resource that supports mental health and well-being.

However, the few studies that have investigated mattering as a mediator between perceived discrimination and well-being have been conducted exclusively with sexual minority populations and have generally treated mattering as a unidimensional construct, failing to explore the potentially distinct contributions of its different forms—such as interpersonal and societal mattering—within this association.

### 1.3. The Current Study

The present study aims to examine the relationship between perceived Islamophobia and social well-being in a group of Muslim women residing in Italy, with particular attention to the psychosocial mechanisms underlying this association. Specifically, the study investigates the mediating role of societal and interpersonal mattering to provide insights into the mechanisms through which Islamophobia may shape well-being outcomes, offering potential directions for interventions aimed at fostering social integration and well-being.

This hypothesis is grounded in the idea that perceived Islamophobia—considered as the subjective perception of being devalued or excluded because of one’s religious identity—may negatively affect individuals’ perceptions of mattering, that is the belief that one is seen as significant and valued by others. Prior literature suggests that discriminatory experiences, and individuals’ perception of being devalued or excluded by others, can foster the internalization of negative social messages, which may lead individuals to feel less acknowledged or appreciated across different areas of social life (Pousson & O’Laughlin, 2024). A lower sense of mattering, in turn, is associated with reduced levels of social well-being, which encompasses a feeling of belonging, integration, and recognition within society (Pousson & O’Laughlin, 2024; C. L. M. Keyes, 1998).

Importantly, mattering is a multidimensional construct. It includes societal mattering (feeling valued by society), mattering to friends (feeling important in peer relationships), and mattering to family (feeling significant within the family context). These domains differ in how they may be related by perceived discrimination and how they contribute to social well-being. For instance, societal mattering may be particularly vulnerable to group-based exclusion, while interpersonal mattering may reflect more immediate relational dynamics that can buffer or intensify the effects of broader stigmatization.

Drawing on these considerations, we predicted that perceived Islamophobia would be directly associated with social well-being among Muslim women (Hypothesis 1). Individuals who perceive higher levels of Islamophobia are expected to report lower levels of social well-being.

Furthermore, we hypothesize that this relationship would be mediated, in distinct ways, by societal mattering, mattering to friends, and mattering to family (Hypothesis 2). While all three dimensions are expected to play a mediating role, we hypothesized differences in the strength of these indirect effects. In particular, we considered societal mattering and mattering to friends as potentially more relevant in this context. Perceived Islamophobia is more likely to be experienced in interactions with institutions, public settings, or peers—contexts in which individuals may report feeling excluded, stereotyped, or devalued because of their religious identity (Abu Khalaf et al., 2023). These domains are more closely associated with perceptions of being acknowledged or dismissed in the broader social environment. In contrast, mattering to family may be less directly associated with perceptions of Islamophobia, as familial relationships often rely on established bonds and shared background, and may not reflect the same exposure to external evaluative pressures. Therefore, while we included all three forms of mattering in the tested model, we hypothesized that societal mattering and mattering to friends would account for a larger share of the indirect effect, whereas mattering to family would exert a smaller mediating effect.

In testing our hypotheses, we considered it important to control for the length of residence in Italy, as previous research has shown that the duration of stay in the host country may influence immigrants’ experiences of discrimination and their overall well-being (Jasinskaja-Lahti et al., 2007; Werkuyten & Nekuee, 1999).

## 2. Materials and Methods

### 2.1. Participants

The study included a total of 120 women who, at the time of data collection, identified as Muslim and were residing in Italy. Participants ranged in age from 18 to 54 years (M = 25.8, SD = 8.59). A detailed breakdown of participants’ nationality, parental background, education, employment, marital status, religiosity, religious practice, and Italian language proficiency is presented in Table 1.

With respect to parental nationality, 40.9% of participants had fathers from other national backgrounds, including Middle Eastern, North African, and South Asian countries, followed by Moroccan (22.5%), Italian (18.3%), Tunisian (12.5%), and Pakistani (5.8%). The distribution of maternal nationality was similar, with 38.3% of participants having mothers from other regions, including Sub-Saharan Africa, the Middle East, and South Asia, followed by Moroccan mothers (24.2%), Italian mothers (20.8%), Tunisian mothers (11.7%), and Pakistani mothers (5.0%).

In terms of education, 42.5% had completed high school, 34.2% held a bachelor’s degree, 12.5% had attained a master’s degree or higher, 8.3% had a middle school diploma, 1.7% had completed primary school, and 0.8% reported other educational qualifications.

Regarding employment status, 34.2% were unemployed, 29.2% worked part-time, 18.3% were seeking their first job, 11.6% were employed full-time, and 6.7% were occasionally employed.

With respect to marital status, 72.5% of the participants were single, 25.0% were married or cohabiting, and 2.5% were separated or divorced. Concerning religiosity, 43.3% identified as fairly religious, 40.8% as very religious, 8.3% as moderately religious, 5.8% as slightly religious, and 1.7% as not religious. Similarly, in terms of religious practice, 51.7% were fairly practicing, 26.7% were very practicing, 14.2% were moderately practicing, 5.8% were slightly practicing, and 1.7% reported not practicing. Finally, with regard to Italian language proficiency, 83.3% of participants reported an advanced understanding of Italian, 80.8% had an advanced reading level, 77.5% had advanced speaking abilities, and 76.7% had advanced writing proficiency.

### 2.2. Measures

Perceived Islamophobia. The Perceived Islamophobia Scale (Kunst et al., 2013) adapted to the Italian context was used to assess the extent to which Muslim individuals perceived general fear of Islam and Muslims, fear of Islamization, and Islamophobia within society. This 12-item scale (e.g., “Many Italians fear an “Islamization” of Italy”) ranges from 1 (definitively disagree) to 6 (definitively agree). Higher scores represented greater levels of perceived Islamophobia. Since this scale was not yet adapted to the Italian context, we tested its factorial structure through CFA and its reliability in terms of internal consistency using both the alpha (α) and the omega (ω) coefficients. CFA confirmed the unidimensional model of the Italian version of the Perceived Islamophobia Scale with a good fit to the data (χ^2^ = 63.8 *p* < 0.007, χ^2^/df = 1.63, RMSEA = 0.07, CFI = 0.97). Also, reliability was good in the present study (α = 0.85, ω = 0.86).

Interpersonal Mattering. Levels of mattering to friends and mattering to family were assessed using the Mattering to Friends subscale and the Mattering to Family subscale, respectively, from the Italian version (Matera et al., 2017) of the Mattering to Others Questionnaire (Marshall, 2001). This scale measures individuals’ perception of being valued within close relationships. Before completing the scale, participants were instructed to reflect on how they believe others perceive them and to select the response that best represented their feelings. The Mattering to Friends subscale consists of 9 items (e.g., “I feel special to my friends”), and the Mattering to Family subscale also includes 9 items (e.g., “My family values me as a person”). Items were rated on a 5-point Likert scale ranging from 1 (Not at all) to 5 (Very much). Higher scores indicate greater levels of mattering to friends (α = 0.96, ω = 96 in the present study) and family (α = 0.93, ω = 94 in the present study), respectively.

Societal Mattering. The Italian version (Paradisi et al., 2023) of the Societal Mattering Scale (Schmidt, 2018) measures individuals’ perception of being important to the broader society. It comprises 9 items (e.g., “The people in the society value me as a person”) with responses on a Likert scale ranges from 1 (Strongly Disagree) to 5 (Strongly Agree). Higher scores indicated greater levels of societal mattering (α = 0.93, ω = 93 in the present study).

Social Well-Being. Social well-being was assessed using the Social Well-Being subscale of the Italian version (Petrillo et al., 2015) of the Mental Health Continuum–Short Form (MHC–SF; C. L. Keyes et al., 2008). This subscale measures individuals’ perceived social well-being and consists of 5 items (e.g., “I felt like I was part of a community, a group or my neighborhood”). Before completing the scale, participants were instructed to respond to the items based on how they had felt during the past month. Responses were rated on a 6-point Likert scale, ranging from 1 (Never) to 6 (Every day). Higher scores indicate greater levels of social well-being (α = 0.85, ω = 86 in the present study).

### 2.3. Procedure

Recruitment was conducted through social media outreach and collaboration with cultural centers across Italy, using a direct and opportunistic sampling approach. Eligibility criteria required participants to (a) be 18 years or older, (b) self-identify as Muslim, and (c) have sufficient proficiency in Italian to complete the survey independently. To ensure cultural appropriateness and relevance, the questionnaire was developed in collaboration with a group of female university students who identified as Muslim and were residing in Italy. Voluntary collaboration (without incentives) was requested from students enrolled at the University of Florence, who contributed to the refinement of the survey. These students participated in a series of pilot sessions in which they reviewed each section of the survey, suggested adaptations in wording, and highlighted potential cultural sensitivities that could influence interpretation. Their feedback led to minor but meaningful revisions (e.g., replacing potentially ambiguous expressions with culturally familiar terms and ensuring inclusivity in references to family and community). This collaborative process enhanced the clarity and contextual sensitivity of the final questionnaire.

Participants were informed that their participation was voluntary, anonymous, and confidential, and that they retained the right to withdraw from the study at any time without justification. Data were collected Via an online questionnaire administered through the Qualtrics^®^ platform (Provo, UT, USA, 2020). The introductory page outlined the study’s objectives, inclusion criteria, confidentiality safeguards, and researcher contact details. Only individuals who confirmed their eligibility and provided informed consent were granted access to the survey, which commenced with socio-demographic questions followed by self-report measures. No financial or material compensation was offered for participation.

Completion of the questionnaire required approximately 15 min. The study received approval from the Ethics Committee of the university with which some of the authors are affiliated (Prot. No. 0090661/2021, with subsequent amendments approved under Minute No. 79/2023). All procedures adhered to the ethical standards of the institutional and/or national research committee, as well as the 1964 Helsinki Declaration and its subsequent amendments or comparable ethical guidelines.

### 2.4. Data Analyses

All statistical analyses were conducted using Jamovi software (The Jamovi Project, 2023; version 2.4.7.0). First, descriptive statistics and Pearson’s correlations among all study variables—perceived Islamophobia, societal mattering, mattering to friends, mattering to family, and social well-being—were computed to examine their distributions and interrelationships.

Second, a mediation model was tested to evaluate the hypothesized relationships. In this model, societal mattering, mattering to friends, and mattering to family were posited as antecedents of social well-being. Perceived Islamophobia was included as a predictor of the three mattering dimensions, while length of residence in Italy was entered as a control variable.

The assumptions for conducting mediation analysis were satisfied (Baron & Kenny, 1986). The hypotheses were tested using the General Linear Model (GLM) package in Jamovi, with bootstrapping (5000 resamples) employed to estimate indirect effects and their bias-corrected confidence intervals (Rucker et al., 2011). This approach provides a robust test of mediation by assessing both the presence and magnitude of indirect effects (Warton et al., 2016).

Effect sizes were reported using standardized beta coefficients (β), and statistical significance was evaluated at *p* = 0.05. Confidence intervals for indirect effects were computed using the percentile bootstrap method, which is recommended for mediation analyses due to its robustness in handling non-normality in indirect effect distributions (Baron & Kenny, 1986; Hayes & Preacher, 2014).

## 3. Results

Table 2 presents the descriptive statistics (means and standard deviations) and intercorrelations among perceived Islamophobia, societal mattering, mattering to friends, mattering to family, and social well-being. The data were normally distributed (skewness < 1.47; kurtosis < 2.82), as skewness values remained below 2 and kurtosis values below 7 (West et al., 1995).

On average, participants reported moderate levels of societal mattering (M = 3.01, SD = 0.96), while mattering to family (M = 4.13, SD = 0.90) and mattering to friends (M = 3.64, SD = 0.99) were relatively higher. Perceived Islamophobia was above the neutral midpoint of the scale (M = 4.54, SD = 0.84), whereas social well-being was slightly below the midpoint (M = 3.26, SD = 1.27).

The correlation analyses revealed significant associations among the study variables. Notably, societal mattering, mattering to friends, and mattering to family were positively and significantly correlated with social well-being (r range = 0.28 to 0.61, all *p*-values < 0.01). Among these, societal mattering showed the strongest positive association with social well-being (r = 0.61, *p* < 0.001), followed by mattering to friends (r = 0.43, *p* < 0.001) and mattering to family (r = 0.28, *p* < 0.001).

Additionally, perceived Islamophobia exhibited a significant negative correlation with social well-being (r = −0.26, *p* < 0.01), indicating that higher levels of perceived Islamophobia were associated with lower social well-being. Perceived Islamophobia was also significantly negatively correlated with mattering to friends (r = −0.20, *p* < 0.05) and societal mattering (r = −0.19, *p* < 0.05). Moreover, societal mattering was positively correlated with both mattering to friends (r = 0.24, *p* < 0.001) and mattering to family (r = 0.20, *p* < 0.05). Mattering to friends and mattering to family were also positively correlated with each other (r = 0.52, *p* < 0.001).

**Figure 1:**
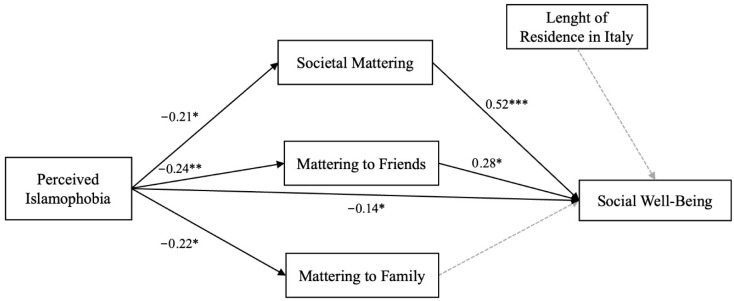
Observed model (*n* = 120). Note. * *p* < 0.05; ** *p* < 0.01; *** *p* < 0.001. Path model illustrates the direct and indirect associations between perceived Islamophobia, mattering dimensions (societal, friends, family), and social well-being. Standardized path coefficients (β) are reported. Solid lines denote significant associations (*p* < 0.05), while dashed lines indicate non-significant paths (*p* > 0.05).

The hypothesized model was tested using Generalized Linear Modeling (GLM) mediation analysis in Jamovi (The Jamovi Project, 2023; version 2.4.7.0) to examine the direct and indirect associations between perceived Islamophobia and social well-being, considering the potential mediating role of societal mattering, mattering to friends, and mattering to family (Figure 1).

Regarding Hypothesis 1, which proposed a direct association between perceived Islamophobia and social well-being, the results indicated that this relationship was marginally significant (*β* = −0.14, *p* = 0.05).

Regarding Hypothesis 2, which posited that the relationship between perceived Islamophobia and social well-being would be indirectly mediated by different dimensions of mattering, the results confirmed the hypothesis for all dimensions of mattering except for mattering to family. Higher levels of perceived Islamophobia were associated with lower levels of societal mattering (*β* = −0.21, *p* < 0.05), mattering to friends (*β* = −0.24, *p* < 0.01), and mattering to family (*β* = −0.22, *p* < 0.05). In turn, societal mattering (*β* = 0.52, *p* < 0.001) and mattering to friends (*β* = 0.28, *p* < 0.05) were positively associated with higher social well-being, whereas mattering to family did not exhibit a significant association (*p* = 0.99).

Concerning the mediation analyses, bootstrapped estimates (5000 resamples) confirmed that perceived Islamophobia was indirectly associated with social well-being through both mattering to friends (indirect effect estimate = −0.102, 95% CI: [−0.231, −0.008], *p* < 0.05) and societal mattering (indirect effect estimate = −0.165, 95% CI: [−0.335, −0.013], *p* < 0.05), supporting Hypothesis 2. However, the indirect association through mattering to family was not statistically significant (indirect effect estimate = 0.0002, 95% CI: [−0.051, 0.071], *p* = 0.99).

Finally, the total association between perceived Islamophobia and social well-being was statistically significant (*β* = −0.32, *p* < 0.001), further highlighting the relevance of both direct and indirect pathways in understanding the relationship between Islamophobia and social well-being. The results of the mediation analyses, including direct, indirect, and total effects, are summarized in Table 3.

## 4. Discussion

The present study examined the associations between perceived Islamophobia and social well-being among Muslim women residing in Italy, exploring the potential mediating role of interpersonal and societal mattering. This study focused on if perceived experiences of Islamophobia may relate to diminished psychological resources, which in turn might be associated with lower social well-being. The focus on social well-being is supported by theoretical perspectives indicating that social dimensions of well-being—such as inclusion and recognition—may be especially relevant in non-Western or marginalized populations (Krys et al., 2019), where individual well-being is often shaped by perceived social value and group belonging. Based on prior research on the psychosocial impact of discrimination, we hypothesized that perceived Islamophobia would be directly and negatively associated with social well-being (Hypothesis 1), and that this association would also be mediated by societal mattering, mattering to friends, and mattering to family (Hypothesis 2).

Consistent with Hypothesis 1, perceived Islamophobia was negatively associated with social well-being. This finding aligns with previous research suggesting that perceived discrimination is associated with lower well-being among members of minority groups (Emmer et al., 2024; Hakim et al., 2018; Stevens & Thijs, 2018). In particular, for Muslim women, who are often exposed to both religious and gendered forms of stigma (Alimahomed-Wilson, 2020; Zine, 2006), the perception of Islamophobia may coincide with experiences of symbolic exclusion, which are negatively associated with their perceived inclusion, recognition, and value as members of society. According to C. L. M. Keyes (1998), social well-being reflects individuals’ perceptions of society and their own role within it; when individuals perceive themselves as devalued or unwelcome in their social environment, this could be associated with lower levels of social well-being. This understanding is consistent with recent cross-cultural evidence suggesting that social well-being may be especially salient in interdependent or collectivistic cultural contexts, where individuals’ sense of well-being is closely tied to social connectedness and group belonging (Krys et al., 2019). From this perspective, for individuals whose cultural identity emphasizes relational harmony and collective recognition—such as many Muslim women—experiences of symbolic exclusion may have particularly detrimental effects on their perceived social integration and well-being. This interpretation is further supported by research on hate crime and Islamophobia, which emphasizes that discriminatory practices operate along a “continuum of hate” (Schweppe & Perry, 2022). Within this framework, subtle forms of exclusion and microaggressions are not isolated from, but structurally linked to, more overt acts of hostility and violence. Understanding Islamophobia as part of this continuum helps to contextualize our findings: even when participants reported primarily symbolic or social forms of exclusion, these experiences may contribute to the same broader processes that undermine belonging and social well-being. In addition, Zaidi and Perry (2025) have shown how Muslims in Canada experience the intersection of visibility, vulnerability, and resilience, highlighting how Islamophobia is lived at the nexus of religious identity, gender, and broader social dynamics. Their analysis of resilience is particularly relevant to the present study, as it underscores the importance of examining not only the detrimental effects of discrimination but also the resources that communities mobilize to preserve well-being despite stigmatization. These perspectives align with our results, showing that social well-being is contingent on how recognition and inclusion are negotiated across multiple levels of social life.

In line with Hypothesis 2, societal mattering and mattering to friends were both significantly related to perceived Islamophobia and social well-being and served as mediators of their association. These findings are consistent with theoretical perspectives that conceptualize mattering as a multidimensional psychological resource that supports well-being, particularly in the context of social marginalization (Prilleltensky, 2020; Flett, 2022). Perceived Islamophobia was associated with lower levels of societal mattering, which in turn was positively associated with social well-being. This finding suggests that when Muslim women perceive widespread societal devaluation of their religious identity, they may be less likely to feel that they are recognized or valued members of the broader social context. According to Flett (2022), societal mattering involves the belief that one’s presence and contributions are appreciated within society. From the perspective of Social Identity Theory (Tajfel & Turner, 1986), group membership plays a central role in shaping one’s self-concept and sense of belonging. When negative societal messages signal that one’s group is not positively valued—as is often the case with Islamophobic discourses—individuals may experience a threat to their social identity, which may be associated with a lower perception of societal mattering. When individuals do not feel like being significant members of the broader society, they might experience lower social well-being, which reflects the perception of being connected to and accepted by society at large (C. L. M. Keyes, 1998).

The indirect association between perceived Islamophobia and social well-being through mattering to friends underscores the role of peer-based validation as a psychological process that may link perceived discrimination to social well-being. These dynamics are consistent with findings showing that peers plays a central role in well-being across collectivistic and minority cultural settings, where strong interpersonal connections help buffer external devaluation (Krys et al., 2019). When Muslim women perceive Islamophobia in their social environment, they may feel less valued or less understood in friendships—particularly when their peers belong to the majority group. From a social identity perspective (Tajfel & Turner, 1986), these perceptions may heighten intergroup awareness and elicit concerns about how one’s identity is viewed in interpersonal contexts. The resulting decline in mattering to friends may reflect not only a reduction in perceived closeness, but also a weakening of the relational bonds through which individuals feel socially anchored.

This lower perception of mattering to friends may, in turn, be associated with lower social well-being, as feeling unacknowledged or unimportant in close relationships can undermine one’s broader sense of social connectedness and inclusion. In line with prior research (Pousson & O’Laughlin, 2024), this pathway suggests that peer affirmation plays a protective role in buffering against the psychological and social correlates of perceived discrimination. Beyond individual-level mechanisms, recent sociological work highlights how belonging and mattering are embedded within broader institutional and minority contexts. Emmerich (2023) documents how language practices and intergenerational dynamics in Turkish mosques in Germany reflect ongoing negotiations of identity and inclusion, shaped both by transnational influences and domestic expectations. Likewise, Emmerich (2024) shows how Jewish–Muslim friendship networks in Germany co-create belonging through shared minority experiences, offering a qualitative perspective on the collective processes that sustain mattering. These insights resonate with the present findings, suggesting that peer validation and societal recognition are not only interpersonal resources but are also situated in institutional and relational fields where minority women navigate visibility, acceptance, and worth.

Moreover, regarding the Hypothesis 2, mattering to family did not mediate the relationship between perceived Islamophobia and social well-being. While perceived Islamophobia was negatively associated with mattering to family—suggesting that higher perceptions of discrimination were linked to lower levels of mattering in the familial domain—this variable was not significantly associated with social well-being in the mediation model. This finding indicates that, although perceived societal devaluation may be associated with how individuals perceive their role and importance within the family, this perception does not appear to be strongly linked to their sense of social well-being when other dimensions of mattering are considered.

One possible interpretation of these findings is that family relationships, while are influenced by broader social dynamics, tend to be grounded in enduring cultural and religious ties that are less contingent on public or institutional recognition. For Muslim women, the family may serve as a culturally coherent space where identity is affirmed, even in the face of perceived societal marginalization. Although mattering to family showed a positive bivariate association with social well-being, it did not account for additional explanatory power in the multivariate model. This result underscores the importance of contextualizing the role of different sources of mattering and suggests that societal and peer-related mattering may be more proximally aligned with social well-being, while familial mattering may relate more closely to other domains, such as emotional or psychological well-being, which were not assessed in this study. This distinction aligns with research suggesting that ingroup-related sources of validation (e.g., family) may be more strongly tied to emotional security, while broader social integration depends on recognition from wider social systems (Krys et al., 2019). Moreover, this interpretation is consistent with recent scholarship on Muslim women’s agency. The introductory chapter of The Cambridge Companion to Women and Islam (Bano, 2025) highlights how agency can be expressed through voluntary adherence to classical gender norms, indicating that familial roles may contribute to meaning and purpose in ways not directly captured by measures of social well-being. Furthermore, the chapter *Becoming Salafi* (Emmerich & Ebbiary, 2025) shows how some Muslim women find empowerment by redefining or even breaking with family traditions. Together, these perspectives provide important context for why mattering to family, although positively associated with well-being at the bivariate level, did not emerge as a significant mediator when compared with societal and peer-related mattering.

Although length of residence in Italy was included as a control variable in the mediation model, it was not significantly associated with social well-being. This suggests that the effects observed in this study are not primarily explained by differences in acculturation time or familiarity with the host context. Rather, the subjective perception of Islamophobia and the sense of mattering appear to exert a role regardless of how long participants have lived in the country. This finding is in line with previous work indicating that perceived discrimination may persist as a salient experience even after extended residence in a host society (Jasinskaja-Lahti et al., 2007).

Overall, these findings contribute to a growing body of research highlighting the role of mattering in understanding how perceived Islamophobia is associated with social well-being. Specifically, they underscore the importance of distinguishing between different forms of mattering, as each may show distinct associations with perceived Islamophobia and with social well-being. In this sample of Muslim women residing in Italy, both societal mattering and mattering to friends were relevant in explaining the negative associations between perceived Islamophobia and social well-being. While mattering to family was not. These findings extend previous work by examining these associations in a European, non-Anglophone context and among a population that remains underrepresented in psychological research. By incorporating these perspectives, the present study situates its findings within broader comparative and sociological discussions of identity, belonging, and women’s agency, thereby providing a richer and more context-sensitive understanding of the mechanisms linking discrimination, mattering, and well-being. At the same time, situating these results within recent contributions on hate crime and Islamophobia (Schweppe & Perry, 2022; Zaidi & Perry, 2025) underscores that the forms of discrimination reported here should not be seen as marginal or isolated. Rather, they are embedded in broader transnational dynamics of exclusion that range from everyday stigmatization to systemic hostility, reinforcing the critical role of societal and peer mattering as protective resources for Muslim women’s well-being.

Despite its contributions, this study presents several limitations that should be acknowledged. First, the cross-sectional design does not allow for causal inferences regarding the associations between perceived Islamophobia, perceived mattering, and social well-being. Future research employing experimental designs is needed to test the causal nature of these relationships and to better understand the psychological mechanisms linking perceived Islamophobia to social well-being. Second, all data were collected through self-report measures, which may be subject to biases such as social desirability or shared method variance. Incorporating multi-method approaches, including qualitative data or observational designs, could provide a more comprehensive understanding of these experiences. Third, while the study focused on Muslim women residing in Italy, the sample was not randomly selected and may not be representative of the broader population of Muslim women in the country. Future studies should aim to recruit more diverse and representative samples in terms of age, region of residence, citizenship status, and generational background.

Moreover, although the present study examined three distinct forms of mattering, other potentially relevant mediators—such as belongingness, public collective self-esteem, or cultural identity—could be explored in future research. It would also be valuable to examine whether protective factors, such as community engagement or positive religious coping, moderate the relationship between perceived Islamophobia and well-being-related outcomes. Finally, given that this research was conducted in a specific national and sociopolitical context, cross-cultural studies are encouraged to determine whether the observed associations hold in other European or non-European settings.

## 5. Conclusions

This study contributes to a more detailed understanding of how perceived Islamophobia is associated with social well-being among Muslim women residing in Italy. The results revealed that societal mattering and mattering to friends were significantly associated with both perceived Islamophobia and social well-being and were identified as mediators of their association. Mattering to family, while negatively associated with perceived Islamophobia and positively correlated with social well-being, did not serve as a mediator. These findings highlight the importance of identifying which dimensions of mattering are most closely linked to experiences of perceived Islamophobia and to individuals’ evaluations of their own social well-being.

Within a community psychology framework, the study highlights the importance of fostering safe environments where Muslim women residing in Italy feel heard and valued, both within their interpersonal networks and within the broader society. In this context, emancipatory and transformative community-based participatory research emerges as a strategic approach to address health and well-being disparities experienced by groups subjected to various forms of oppression (Suarez-Balcazar, 2020), such as Muslim women. This strengths-based approach promotes the co-creation of knowledge by community members and supports culturally relevant interventions (Suarez-Balcazar, 2020). It has the potential to generate meaningful social change (Suarez-Balcazar, 2020), reinforcing the perception of societal mattering as well as the visibility and recognition of Muslim women in institutional and civic life. Future interventions might also focus on enhancing mattering to friends by facilitating inclusive peer interactions, particularly in settings where Muslim women might otherwise feel marginalized due to religious or cultural identity. From a social psychology perspective, the findings reinforce the significance of perceived social value and recognition in shaping how minority individuals experience their position within society.

Taken together, these findings suggest that enhancing societal mattering and mattering to friends—through targeted policies, educational initiatives, and culturally sensitive practices—may represent effective strategies to support the social well-being of Muslim women in contexts where Islamophobia is perceived. Interventions based on intergroup contact theory, identity-affirming practices, and the reduction in symbolic exclusion may help counteract the negative associations identified in this study. Promoting these dimensions of mattering can be a key component in addressing social marginalization and in fostering more cohesive, equitable communities.

## Figures and Tables

**Table 1:** Sociodemographic characteristics of the sample (*n* = 120).

Variable	Category	%
Age	Range: 18–54 years (M = 25.8, SD = 8.59)	—
Nationality	Italian citizens	45.1
	Moroccan	5.8
	Pakistani	1.7
	Tunisian	1.7
	Iraqi	1.6
	Ivorian	1.6
	Afghan	0.8
	Jordanian	0.8
	Senegalese	0.8
	Burkinabe	0.8
	Bengali	0.8
	Syrian	0.8
	Algerian	0.8
	Mixed Italian–Arab	6.7
	Other (Egypt, Lebanon, Turkey, Iran, Somalia, Sudan, Indonesia, other Middle Eastern, North African, and South Asian countries)	29.2
Parental nationality (fathers)	Other regions (Middle East, North Africa, South Asia, Sub-Saharan Africa)	40.9
	Moroccan	22.5
	Italian	18.3
	Tunisian	12.5
	Pakistani	5.8
Parental nationality (mothers)	Other regions (Middle East, North Africa, South Asia, Sub-Saharan Africa)	38.3
	Moroccan	24.2
	Italian	20.8
	Tunisian	11.7
	Pakistani	5.0
Education	Primary school	1.7
	Middle school	8.3
	High school	42.5
	Bachelor’s degree	34.2
	Master’s degree or higher	12.5
	Other	0.8
Employment status	Unemployed	34.2
	Part-time	29.2
	Seeking first job	18.3
	Full-time	11.6
	Occasional	6.7
Marital status	Single	72.5
	Married/cohabiting	25.0
	Separated/divorced	2.5
Religiosity	Very religious	40.8
	Fairly religious	43.3
	Moderately religious	8.3
	Slightly religious	5.8
	Not religious	1.7
Religious practice	Very practicing	26.7
	Fairly practicing	51.7
	Moderately practicing	14.2
	Slightly practicing	5.8
	Not practicing	1.7
Italian proficiency	Advanced understanding	83.3
	Advanced reading	80.8
	Advanced speaking	77.5
	Advanced writing	76.7

**Table 2:** Descriptive statistics and bivariate correlations among the variables of interest (*n* = 120).

	1	2	3	4	5	M (SD)
1. Perceived Islamophobia	1					4.54 (0.84)
2. Societal mattering	−0.19 *	1				3.01 (0.96)
3. Mattering to Friends	−0.20 *	0.24 **	1			3.64 (0.99)
4. Mattering to Family	−0.15	0.20 *	0.52 ***	1		4.13 (0.90)
5. Social Well-being	−0.26 **	0.61 ***	0.43 ***	0.28 **	1	3.26 (1.27)

Note. * *p* < 0.05; ** *p* < 0.01; *** *p* < 0.001.

**Table 3:** Mediation analysis results of perceived Islamophobia on social well-being Via societal, friends, and family mattering (*n* = 120). Standardized coefficients (*β*) reported.

Effect Type	Path	*β*	*p*	95% CI
Direct effect	Perceived Islamophobia → Social well-being	−0.14	0.05	–
Indirect effect	Perceived Islamophobia → Societal mattering → Social well-being	−0.165	0.05	[−0.335, −0.013]
	Perceived Islamophobia → Mattering to friends → Social well-being	−0.102	0.05	[−0.231, −0.008]
	Perceived Islamophobia → Mattering to family → Social well-being	0.0002	0.99	[−0.051, 0.071]
Total effect	Perceived Islamophobia → Social well-being	−0.32	<0.001	–

## Data Availability

The data are not publicly available but are held by the corresponding author and can be provided upon reasonable request and justified purpose. The dataset is fully anonymous.
